# Strict-Feedback Backstepping Digital Twin and Machine Learning Solution in AE Signals for Bearing Crack Identification

**DOI:** 10.3390/s22020539

**Published:** 2022-01-11

**Authors:** Farzin Piltan, Rafia Nishat Toma, Dongkoo Shon, Kichang Im, Hyun-Kyun Choi, Dae-Seung Yoo, Jong-Myon Kim

**Affiliations:** 1 Department of Electrical, Electronics and Computer Engineering, University of Ulsan, Ulsan 44610, Korea; piltanfarzin@gmail.com (F.P.); rafiatoma.eceku@gmail.com (R.N.T.); dongkoo88@gmail.com (D.S.); 2 ICT Convergence Safety Research Center, University of Ulsan, Ulsan, 44610, Korea; kichang@ulsan.ac.kr; 3 Electronics and Telecommunications Research Institute (ETRI), Daejeon 34129, Korea; choihk@etri.re.kr (H.-K.C.); ooseyds@etri.re.kr (D.-S.Y.)

**Keywords:** bearing, digital twin, machine learning, acoustic emission, autoregressive technique, Gaussian process regression, Laguerre filter, fuzzy logic, strict-feedback backstepping observer, support vector regression, support vector machine, crack size diagnosis, crack type diagnosis

## Abstract

Bearings are nonlinear systems that can be used in several industrial applications. In this study, the combination of a strict-feedback backstepping digital twin and machine learning algorithm was developed for bearing crack type/size diagnosis. Acoustic emission sensors were used to collect normal and abnormal data for various crack sizes and motor speeds. The proposed method has three main steps. In the first step, the strict-feedback backstepping digital twin is designed for acoustic emission signal modeling and estimation. After that, the acoustic emission residual signal is generated. Finally, a support vector machine is recommended for crack type/size classification. The proposed digital twin is presented in two steps, (a) AE signal modeling and (b) AE signal estimation. The AE signal in normal conditions is modeled using an autoregressive technique, the Laguerre algorithm, a support vector regression technique and a Gaussian process regression procedure. To design the proposed digital twin, a strict-feedback backstepping observer, an integral term, a support vector regression and a fuzzy logic algorithm are suggested for AE signal estimation. The Ulsan Industrial Artificial Intelligence (UIAI) Lab’s bearing dataset was used to test the efficiency of the combined strict-feedback backstepping digital twin and machine learning technique for bearing crack type/size diagnosis. The average accuracies of the crack type diagnosis and crack size diagnosis of acoustic emission signals for the bearings used in the proposed algorithm were 97.13% and 96.9%, respectively.

## 1. Introduction

Bearings are very important components in rotating machines, as they are used to reduce the friction between moving parts for linear and rotational motion. These components have been widely used in rotating machinery in various industries, such as steel mills, paper mills and wind power generators, to improve their lifespan and efficiency by reducing friction and facilitating motion. Due to the widespread use of these components, complexities of the tasks and nonlinear parameters, it is particularly significant to investigate their associated faults. Different types of faults have been introduced in the bearing which can be categorized into four main groups, inner fault, outer fault, roller fault and cage faults [1,2].

To analyze the bearing conditions, several condition monitoring procedures based on acoustic emissions, bearing circuit analysis, stator current, vibration, shaft voltage and bearing current have been considered [1,2]. The types of sensors and crack diagnosis approaches in bearings can be quite different, depending on the projects and facilities. Among these, vibration and acoustic emission measurement techniques have been the most widely used [2]. 

Multiple techniques exploring the time domain, frequency domain and time–frequency domain features have been carried out to design bearing fault diagnosis schemes. In feature-based analyses, time and frequency domain analysis has the challenge of a high-dimensional feature set. On the other hand, the analysis of the bearing signals by classical signal processing, such as the fast Fourier transform, is considered to be insufficient, because it provides a global transformation that is unable to properly capture the local time–frequency properties of a signal [3]. The nonlinear and nonstationary behavior in bearing can be explored by various time–frequency analysis techniques, including the Wigner Ville distribution (WVD) [4], short-time Fourier transform (STFT) [5,6] and wavelet packet transform (WPT) [7].

Crack diagnosis approaches can be characterized into three main divisions, classical techniques (e.g., model-reference approach), intelligent approaches (data-driven approach) and a combination of classical and intelligent algorithms (hybrid approach) [1]. The model-reference and data-driven approaches have various benefits and disadvantages [8]. The biggest issues of model-reference schemes are model dependence and low efficiency in uncertain environments [8]. On the other hand, robustness and reliability are two essential deficiencies of data-driven techniques [9]. Recently, a lot of attention has been paid to hybrid techniques for fault diagnosis. These techniques can increase the reliability and accuracy of fault identification through a combination of other procedures [10]. To reduce the obstructions of model-reference and data-driven algorithms, heterogeneous schemes have been suggested [11]. The combination of feature extraction in AE signals and support vector machines was introduced in [12]. Moreover, Junayed Hassan et al. [13] suggested the combination of AE feature extraction, genetic algorithm-based feature selection and k-nearest neighbor (k-NN) classifier for fault diagnosis.

Digital twins are an emerging technique that can be used in anomaly diagnosis. These methods allow various and much more detailed analyses to be performed by designing digital models of the system [14]. Digital twins have many uses, but the most notable ones are modeling and estimation. Various techniques can be selected for system modeling, including mathematical and data-driven approaches [14]. 

Recently, many articles have represented data-driven methods for system modeling. The main principle of most data-driven modeling techniques is regression. The application of system modeling using an autoregressive external input with the Laguerre technique was presented in [15]. Moreover, to improve the performance of system/signal modeling using the autoregressive technique, Zhou Yihong and Feng Ding [16] represented the combination of an autoregressive technique and radial basis function. The application of nonlinear autoregressive techniques for modeling systems controlled using an intelligent Kalman filter approach are explained in [17]. Linear regressors can provide acceptable results for modeling stationary signals, but they have many limitations for non-stationary signals such as vibration or acoustic emission (AE) signals. Thus, to modeling vibration signals, Tayebihaghighi and Koo [18] introduced the machine learning-based autoregressive technique. To improve the robustness and reliability of signal modeling, a signal estimation technique using observation techniques has been proposed. Various kinds of observers have been used in several applications and can be categorized into two main groups, linear observers (e.g., proportional integral observer, proportional multi-integral observer and proportional integral derivative observer) and nonlinear observers (e.g., sliding mode observer, feedback linearization observer, backstepping observer, fuzzy logic observer and neural network observer) [19]. Apart from the applications of linear observers for fault diagnosis, these techniques suffer from reliability, robustness and nonlinear signal estimation issues [15]. Njima and Garna [15] improved the performance of proportional integral observers using an autoregressive external input with the Laguerre technique. To improve linear observers, feedback linearization observers were introduced in [20].

The main issue associated with this approach is robustness. To solve this robustness problem in feedback linearization observers, sliding mode observers and backstepping observers have been suggested [21,22]. The chattering phenomenon is one of the main critical challenges of a classical siding mode observer. On the other hand, reliability is the main issue for fuzzy and neural network observers [23,24,25,26]. Moreover, the application of fuzzy logic approaches for fault classification of bearings is presented in [27]. To improve the reliability of fuzzy techniques, global fuzzy entropy with fuzzy-based classifier was introduced in [28]. 

Diverse algorithms have been recommended to accomplish the decision-making procedure, which are distributed into two principal groups, (a) classical techniques and (b) machine learning-based approaches. Classical methods such as rule-based algorithms have been widely used for decades in different expert systems [29]. On the other hand, machine learning algorithms for decision making are currently more frequently used for condition monitoring in nonlinear and nonstationary systems/signals. The most popular algorithms for classifying the signals are the support vector machine (SVM) [30], decision tree [31], k-nearest neighbors [32], naive bayes [33] and logistic regression [34]. The SVM is known as a robust machine learning algorithm that is impervious to the curse of the dimensionality problem. One of the main advantages of the SVM is that it can be proficiently applied for the classification of both linear and nonlinear separable types of datasets, which is possible due to the availability of different types of kernels, such as linear, polynomial and radial basis function kernels [30]. In this work, an intelligent signal identification algorithm for modeling, hybrid-based AE signal observation and the support vector machine (SVM) classification method are recommended for bearing crack type/size diagnosis. In the proposed algorithm (digital twin), first, the AE signal in normal condition is modelled using the combination of proposed linear regression, a nonlinear regressor and a Laguerre filter. Next, the unknown AE signals are estimated using the proposed digital twin. Finally, the SVR is used for crack type and crack size diagnosis. 

This work has the following contributions:

1. AE signal modeling using a combination of autoregressive techniques, Laguerre filters, support vector regression and Gaussian process regression.
2. Design of a strict-feedback backstepping digital twin using the proposed signal modeling, strict-feedback backstepping observer, integral term, support vector machine and fuzzy algorithm for normal and abnormal AE signal estimation.
3. Proposal of a digital twin and machine learning algorithm for crack type/size diagnosis.

This work is organized as follows: The acoustic emission bearing dataset is explained in Section 2. The strict-feedback backstepping digital twin, AE residual signal generation and crack type/size classification using the SVM are presented in Section 3. The results and discussion are analyzed and explained in Section 4. Finally, conclusions and future works are in Section 5.

## 2. Dataset

**Figure 1:**
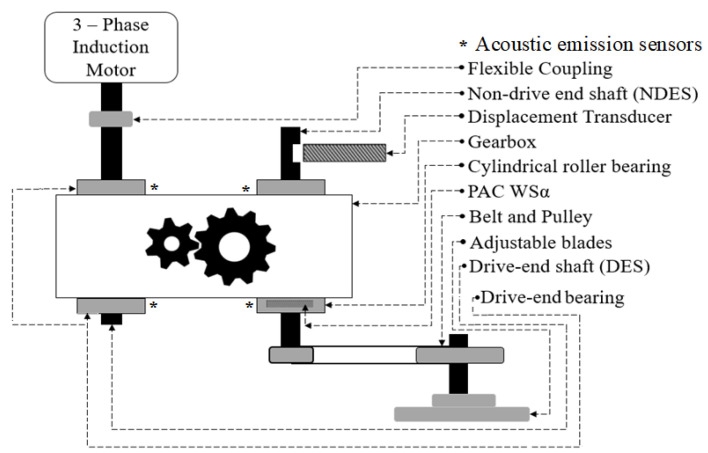
Schematic of the fault simulator.

**Figure 2:**
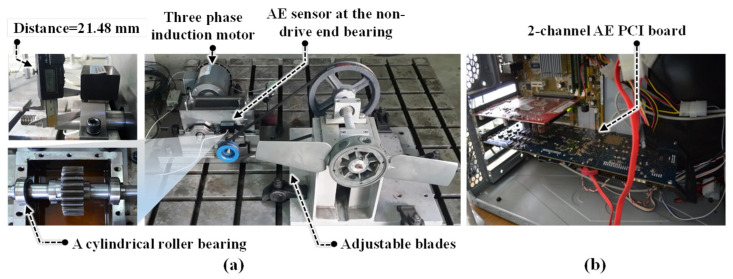
Experimental setup for bearing data collection: (**a**) recording the data and (**b**) acoustic emission data acquisition.

Figure 1 illustrates a block of the fault simulator. A three-phase induction motor is suggested to transfer the torque to the non-drive end shaft using a gearbox. Figure 2 illustrates an Ulsan Industrial Artificial Intelligence (UILI) Lab testbed for the simulation of the bearing faults.

**Figure 3:**
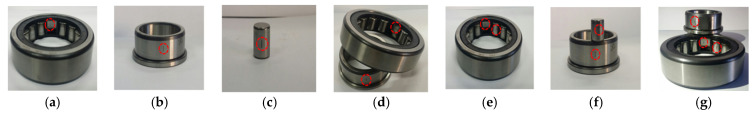
State of the bearing: (**a**) OC, (**b**) IC, (**c**) BC, (**d**) IOC, (**e**) IBC, (**f**) OBC and (**g**) IOBC.

This testbed had the following parts: (a) a three-phase induction motor; (b) a gearbox, to transfer the load to the shaft; and (c) acoustic emission sensors for data collection. For each shaft, a bearing (FAG NJ206-E-TVP2) was attached. In this experiment, a wideband frequency AE sensor (PAC WSα) was used to acquire data when placed on the top of non-drive end shaft bearing at a 21.48 mm displacement [35,36,37,38]. In this paper, crack sizes in the bearings 3 mm and 6 mm in length, 0.35 mm in width and 0.3 mm in depth were tested [35,39,40]. Furthermore, the following eight different conditions to test the bearing were introduced: healthy conditions (HC), ball conditions (BC), inner conditions (IC), outer conditions (OC), inner–ball conditions (IBC), inner–outer conditions (IOC), outer–ball conditions (OBC) and inner–outer–ball conditions (IOBC). Figure 3 shows the bearing crack types. 

Moreover, the sampling rate to collect the data was 250 kHz. Fault diagnosis of low-speed bearings under variable speed conditions is still a challenging problem and the motor rotational speeds were 300, 400, 450 and 500 RPM. Table 1 shows the AE signal information. Moreover, Table 2 illustrates the detail of the data acquisition system [36].

## 3. Proposed Scheme

**Figure 4:**
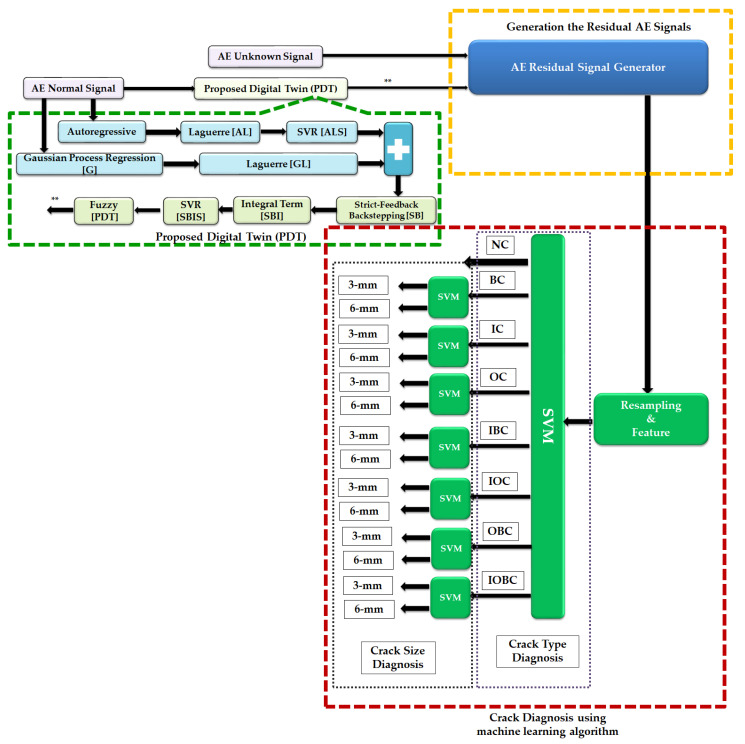
Structure of the proposed digital twin with machine learning for crack type/size diagnosis. **: The output of the proposed digital twin.

Figure 4 illustrates the proposed technique for crack diagnosis in bearings using AE signals. Based on this figure, the proposed method has the following three main parts: (a) proposed digital twin technique for AE signal modeling and estimation, (b) generation of residual AE signals and (c) crack type/size diagnosis using a machine learning algorithm (support vector machine). To design the proposed digital twin algorithm, the main step is signal estimation. In this work, a proposed hybrid robust observation algorithm was designed for estimating the AE signals. To extract the state-space function from AE signals to design the proposed hybrid robust observer, signal approximation (modeling) is the first step. A nonlinear function of support vector regression (SVR) and the nonlinear Gaussian process regression (GPR) are applied to the robust autoregressive Laguerre to have a robust and accurate hybrid AE signal modeling algorithm. After modeling the AE signal in normal conditions, the hybrid modeling algorithm and fuzzy logic approaches are applied to the proposed strict-backstepping observer to design a proposed digital twin for AE signal estimation. According to Figure 4, after the digital twin is designed in the first step, the AE residual generation, which is the difference between the original and the estimation signal, is obtained in the second stage. Finally, in the last stage, the crack size can be identified using a support vector machine (SVM). So, first, the AE residual signal is resampled and the root-mean-square (RMS) feature is extracted from AE signals. Then, the RMS of the residual signal is identified by SVM classification algorithms.

### 3.1. Proposed Digital Twin for Signal Modeling and Estimation

To design the proposed digital twin algorithm, the main step is signal estimation. In this work, a proposed hybrid robust observation algorithm was designed for estimating the AE signals. To extract the state-space function from AE signals to design the proposed hybrid robust observer, signal approximation (modeling) is the first step. This section has two sub-sections. First, the design of AE signal modeling in normal conditions based on the proposed hybrid technique is explained. After that, the design of the proposed hybrid observer to estimate the AE signals is presented. 

#### 3.1.1. Proposed Signal Modeling Using ALS-GL Algorithm

To model the AE signal, a proposed Gaussian support vector autoregressive Laguerre technique (ALS-GL) was designed. So, based on Figure 4, first, the autoregressive algorithm was developed for AE signal extraction. After that, the Laguerre technique was applied to the autoregressive method (AL) to improve the robustness. Next, the support vector regression was designed with autoregressive Laguerre (ALS) to have a nonlinear regressor. Finally, the Gaussian process regression Laguerre was designed with the support vector autoregressive Laguerre (ALS-GL) to reduce the error and develop the proposed AE signal modeling. The autoregressive technique is described by Equation (1).

(1) $$\{\begin{array}{l} \Psi_{A}\left(k+1\right)=\left[\sigma_{\Psi }\Psi_{A}\left(k\right)+\sigma_{i}\Phi_{i}\left(k\right)\right]+e_{A}\left(k\right)+\varphi \left(k\right)\\ H_{A}\left(k\right)={\left(\sigma_{o}\right)}^{T}\Psi_{A}\left(k\right) \end{array}$$

where $\Psi_{A}\left(k\right),\Phi_{i}\left(k\right),e_{A}\left(k\right),\varphi \left(k\right),\;H_{A}\left(k\right)$ and $\left(\sigma_{\Psi },\sigma_{i},\sigma_{o}\right)$ are the state of the AE signal in normal conditions using the autoregressive technique, the measurable AE signal, the error for signal modeling using the autoregressive approach, the unknown (uncertain) conditions in normal states, the output state of the AE signal in normal conditions using the autoregressive technique and the coefficients (state, measurable AE signal and output state of the AE signal), respectively. The error of signal modeling using the autoregressive approach was generated using the following definition:

(2) $$e_{A}\left(k\right)=H_{A}\left(k\right)-H_{A}\left(k-1\right)$$

To improve the robustness of autoregressive AE signal modeling, the Laguerre filter was combined with the autoregressive technique (referred to as AL) and was introduced using the following definition:

(3) $$\{\begin{array}{l} \Psi_{AL}\left(k+1\right)=\left[\sigma_{\Psi }\Psi_{AL}\left(k\right)+\sigma_{i}\Phi_{i}\left(k\right)+\sigma_{H}H_{AL}\left(k\right)\right]+e_{AL}\left(k\right)+\varphi \left(k\right)\\ H_{AL}\left(k\right)={\left(\sigma_{o}\right)}^{T}\Psi_{AL}\left(k\right) \end{array}$$

where $\Psi_{AL}\left(k\right),e_{AL}\left(k\right),\;H_{AL}\left(k\right)$ and $\sigma_{H}\;$ are the state of the AE signal in normal conditions using the AL technique, the error of signal modeling using the AL approach, the output state of the AE signal in normal conditions using the AL technique and a coefficient, respectively. Moreover, the error of signal modeling using AL approach was generated via the following description:

(4) $$e_{AL}\left(k\right)=H_{AL}\left(k\right)-H_{AL}\left(k-1\right)$$

To reduce the effects of complexity and nonlinearity of the bearing behavior, the nonlinear technique using a support vector regression (SVR) is suggested. Thus, the combination of the AL technique and SVR (referred to as ALS) is introduced by the following definition:

(5) $$\{\begin{array}{l} \Psi_{ALS}\left(k+1\right)=\left[\sigma_{\Psi }\Psi_{ALS}\left(k\right)+\sigma_{i}\Phi_{i}\left(k\right)+\sigma_{H}H_{ALS}\left(k\right)+\sigma_{SVR}H_{SVR}\left(k\right)\right]+e_{ALS}\left(k\right)\\ +\varphi \left(k\right)\\ H_{ALS}\left(k\right)={\left(\sigma_{o}\right)}^{T}\Psi_{ALS}\left(k\right) \end{array}$$

where $\Psi_{ALS}\left(k\right),e_{ALS}\left(k\right),\;H_{ALS}\left(k\right),\;H_{SVR}$ and $\sigma_{SVR}\;$ are the state of the AE signal in normal conditions using the ALS technique, the error of signal modeling using the ALS approach, the output state of the AE signal in normal conditions using the ALS technique, the output state of the AE signal in normal conditions using the SVR method and a coefficient, respectively. Furthermore, the error of signal modeling using the ALS approach is given by the following equation:

(6) $$e_{ALS}\left(k\right)=H_{ALS}\left(k\right)-H_{ALS}\left(k-1\right)$$

The SVR technique is a learning-based algorithm that can be used for signal approximation. The nonlinear regression function using the kernel trick is given by the following definition:

(7) $$H_{SVR}=\sum_{i}\left(\varrho_{i}{}^{+}-\varrho_{i}{}^{-}\right)K\left(\phi_{i},\phi \right)+\beta$$

where $H_{SVR},\left(\varrho_{i}{}^{+}-\varrho_{i}{}^{-}\right),K\left(\phi_{i},\phi \right)$ and β are the output state of the AE signal in normal conditions using the SVR method, the Lagrange coefficients in the SVR function, the kernel of the SVR function and the bias of the SVR function, respectively. The Gaussian kernel function is defined as follows:

(8) $$K\left(\phi_{i},\phi \right)=e^{\left(-\frac{1}{2\varepsilon^{2}}||\phi_{i}-\phi {\left|\right|}^{2}\right)}$$

where ε is the variance of the signal. Additionally, to minimize $\left(\varrho_{i}{}^{+}-\varrho_{i}{}^{-}\right)$, we have

(9) $$\min\sum_{i}\sum_{j}\left(\varrho_{i}{}^{+}-\varrho_{i}{}^{-}\right)\left(\varrho_{j}{}^{+}-\varrho_{j}{}^{-}\right)\Theta_{ij}$$

where $K\left(\phi_{i},\phi \right)$ is defined by $\Theta_{ij}.$ So, Equation (9) can be re-written as follows:

(10) $$\min\sum_{i}\sum_{j}\varrho_{i}{}^{+}\varrho_{j}{}^{+}\Theta_{ij}-\varrho_{i}{}^{-}\varrho_{j}{}^{+}\Theta_{ij}-\varrho_{i}{}^{+}\varrho_{j}{}^{-}\Theta_{ij}+\varrho_{i}{}^{-}\varrho_{j}{}^{-}\Theta_{ij}$$

If $\Theta =\left[\Theta_{ij}\right]\in {\mathbb{R}}^{n\times n}$, $\varrho ={\left[\begin{array}{c} \varrho^{+}\\ \varrho^{-} \end{array}\right]}_{2n\times 1}$ and $\varpi =\left[\begin{array}{cc} \Theta & -\Theta \\ -\Theta & \Theta \end{array}\right]$, we have

(11) $$\min0.5\varrho^{T}\varpi \varrho +\Delta^{T}\varrho$$

where $\Delta ={\left[\begin{array}{c} -H+\rho \\ H+\rho \end{array}\right]}_{2n\times 1}$, H and ρ are the output state of the AE signal and accepted boundary layer (error) of signal modeling, respectively. Furthermore, the bias of the SVR function can be introduced as the following function:

(12) $$\beta =\frac{1}{\left|S\right|}\underset{s\in S}{\sum }\left[\begin{array}{l} H_{s}-\sum_{i\in S}\left(\varrho_{i}{}^{+}-\varrho_{i}{}^{-}\right)\times \\ K\left(\phi_{i},\phi \right)-\rho \\ \times sgn\left(\varrho_{i}{}^{+}-\varrho_{i}{}^{-}\right) \end{array}\right]$$

where $H_{s}$ and S are the output support vector and support vector, respectively. In addition, the support vector, S, is introduced by the following definition:

(13) $$S=\left\{i|0<\varrho_{i}{}^{+}+\varrho_{i}{}^{-}<\eta \right\}$$

where η is an upper band constant. The Vapnik loss function (VLF) was defined to determine the signal modeling accuracy based on the SVR.

(14) $$V_{\in }\left(H_{SVR},H\right)=\{\begin{array}{c} 0\\ for:\left|H_{SVR}-H\leq \rho \right|\\ \left|H_{SVR}-H\right|-\rho \\ for:\mathrm{Otherwise} \end{array}$$

The bearing is a nonlinear system. Moreover, extracting the state-space function from AE signals is very difficult. In the second step, the combination of the Gaussian process regression (GPR) algorithm and Laguerre technique (referred to as GL) is recommended. To increase the accuracy and improve the reliability of AE signal modeling, the ALS approach was combined with the GL technique, referred to as ALS-GL. The GPR is a nonlinear approach for function approximation based on nonlinear kernels. The state-space function using the GPR technique is described by Equation (15).

(15) $$\{\begin{array}{l} \Psi_{G}\left(k+1\right)=\left[\chi_{G}\Psi_{G}\left(k\right)+\Upsilon_{i}\Phi_{i}\left(k\right)\right]+e_{G}\left(k\right)\\ +\varphi \left(k\right)\\ H_{G}\left(k\right)={\left(\Upsilon_{o}\right)}^{T}\left(\omega_{n}\right)\chi_{G}{}^{-1}\Psi_{G}\left(k\right) \end{array}$$

where $\Psi_{G}\left(k\right),\chi_{G},\Phi_{i}\left(k\right),e_{G}\left(k\right),\varphi \left(k\right),\;H_{G}\left(k\right)$ and $\left(\Upsilon_{i},\Upsilon_{o}\right)$ are the state of the AE signal in normal conditions using the GPR technique, the covariance matrix for the AE signal in normal conditions using the GPR technique, the measurable AE signal, the error of signal modeling using the GPR approach, the unknown (uncertain) conditions in normal states, the output state of the AE signal in normal conditions using the GPR technique and coefficients (measurable AE signal and output state of AE signal), respectively. Additionally, the error of signal modeling using the GPR approach was generated using the following definition:

(16) $$e_{G}\left(k\right)=H_{G}\left(k\right)-H_{G}\left(k-1\right)$$

Moreover, the covariance matrix for the AE signal in normal conditions using the GPR technique is introduced by the following definition:

(17) $$\chi_{G}=\gamma^{2}e^{\left(-0.5\Psi_{G}{}^{T}W^{-1}\Psi_{G}\right)}+\vartheta$$

(18) $$W=diag{\left(T\right)}^{2}$$

where γ,ϑ and T, respectively, are the variance in the AE signal in a normal state, the variance in noise and the width of the kernel. To improve the robustness of the GPR algorithm, the combination of the GPR and Laguerre methods, hereafter called GL, is introduced by the following equation:

(19) $$\{\begin{array}{l} \Psi_{GL}\left(k+1\right)=\left[\chi_{GL}\Psi_{GL}\left(k\right)+\Upsilon_{i}\Phi_{i}\left(k\right)+\Upsilon_{H}H_{GL}\left(k\right)\right]+e_{GL}\left(k\right)+\varphi \left(k\right)\\ H_{GL}\left(k\right)={\left(\Upsilon_{o}\right)}^{T}\left(\omega_{n}\right)\chi_{GL}{}^{-1}\Psi_{GL}\left(k\right) \end{array}$$

where $\Psi_{GL}\left(k\right),\chi_{GL},e_{GL}\left(k\right),\;H_{GL}\left(k\right)$ and $\left(\Upsilon_{H}\right)$ are the state of the AE signal in normal conditions using the GL technique, the covariance matrix for the AE signal in normal conditions using the GL method, the error of signal modeling using the GL approach, the output state of the AE signal in normal conditions using the GL technique and a coefficient, respectively. Furthermore, the error of signal modeling using the GL method is given by the following definition:

(20) $$e_{GL}\left(k\right)=H_{GL}\left(k\right)-H_{GL}\left(k-1\right)$$

Moreover, the covariance matrix for the AE signal in normal conditions using the GL technique is given by the following definition:

(21) $$\chi_{GL}=\gamma^{2}e^{\left(-0.5\Psi_{GL}{}^{T}W^{-1}\Psi_{GL}\right)}+\vartheta$$

To improve the accuracy, robustness and reliability of bearing modeling, the ALS approach and GL method are suggested for AE signal in normal conditions, as described by the following equation:

(22) $$\{\begin{array}{l} \Psi_{ALSGL}\left(k+1\right)=\Psi_{ALS}\left(k\right)+\Psi_{GL}\left(k\right)\\ H_{ALSGL}\left(k\right)=H_{ALS}\left(k\right)+H_{GL}\left(k\right) \end{array}$$

Consequently, we have

(23) $$\{\begin{array}{l} \Psi_{ALSGL}\left(k+1\right)=\left[\chi_{\mathrm{ALSGL}}\Psi_{ALSGL}\left(k\right)+\Upsilon_{i}\Phi_{i}\left(k\right)+\Upsilon_{H}H_{ALSGL}\left(k\right)\right]+e_{ALSGL}\left(k\right)+\varphi \left(k\right)\\ H_{ALSGL}\left(k\right)={\left(\Upsilon_{o}\right)}^{T}\left(\omega_{n}\right)\chi_{\mathrm{ALSGL}}{}^{-1}\Psi_{ALSGL}\left(k\right) \end{array}$$

Moreover, the error of system modeling using the proposed ALS-GL approach was calculated using the following equation:

(24) $$e_{ALSGL}\left(k\right)=H_{ALSGL}\left(k\right)-H_{ALSGL}\left(k-1\right)$$

**Figure 5:**
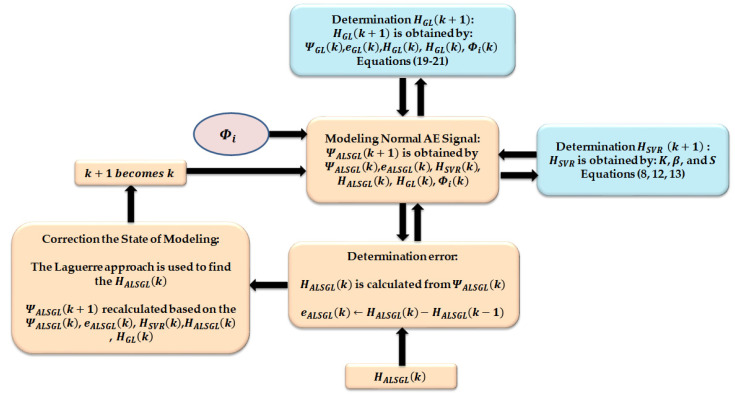
Flow chart typical of AE signal modeling using ALS-GL approach.

The covariance matrix for the AE signal in normal conditions using the ALS-GL technique is introduced by the following definition:

(25) $$\chi_{\mathrm{ALSGL}}=\gamma^{2}e^{\left(-0.5\Psi_{ALSGL}{}^{T}W^{-1}\Psi_{ALSGL}\right)}+\vartheta$$

where $\Psi_{ALSGL}\left(k\right),\chi_{ALSGL},e_{ALSGL}\left(k\right),$ and $H_{ALSGL}\left(k\right)$ are the state of the AE signal in normal conditions using the proposed ALS-GL technique, the covariance matrix for the AE signal in normal conditions using the ALS-GL method, the error of signal modeling using the ALS-GL approach and the output state of the AE signal in normal conditions using the ALS-GL technique, respectively. Figure 5 shows the flow chart typical of AE signal modeling using the proposed ALS-GL technique. 

#### 3.1.2. Proposed Signal Estimation Using Hybrid Algorithm

Apart from the accuracy of the proposed bearing modeling, it has limitations for uncertain conditions. To address this issue, a digital twin (PDT) was designed in this work. After modeling the AE signal in normal conditions, based on Figure 4, the hybrid modeling algorithm and fuzzy logic approaches were applied to the proposed strict-backstepping observer to design a proposed digital twin for AE signal estimation. To design the proposed digital twin, first, the proposed hybrid AE signal modeling algorithm (ALS-GL) was applied to the nonlinear robust strict-feedback backstepping observer (SBO). After that, the integral term was applied to the strict-feedback backstepping (SBI) observer to reduce the signal estimation error. Finally, in the last part of the proposed digital twin design, the fuzzy logic approach was applied to the strict-feedback integral backstepping observer to improve the effect of uncertainty estimation. Thus, the classical strict-feedback backstepping observer (SBO) is defined by the following equation:

(26) $$\{\begin{array}{l} \Psi_{ALSGL\text{-}SB}\left(k+1\right)=[\chi_{\mathrm{ALSGL}}\Psi_{ALSGL\text{-}SB}\left(k\right)\\ +\Upsilon_{H}(H_{ALSGL\text{-}SB}\left(k\right)+Ζ\left(k\right)]+e_{ALSGL\text{-}SB}\left(k\right)\\ \;\;\;\;\;\;+\varphi_{SB}\left(k\right)+\Upsilon_{i}\psi_{SB}\Phi_{i}\left(k\right)\\ H_{ALSGL\text{-}SB}\left(k\right)={\left(\Upsilon_{o}\right)}^{T}\left(\omega_{n}\right)\chi_{\mathrm{ALSGL}}{}^{-1}\Psi_{ALSGL\text{-}SB}\left(k\right) \end{array}$$

where $\Psi_{ALSGL\text{-}SB}\left(k\right),e_{ALSGL\text{-}SB}\left(k\right),Ζ\left(k\right),\varphi_{SB}\left(k\right),\psi_{SB}$ and $H_{ALSGL\text{-}SB}\left(k\right)$ are the state estimation of AE signals using the *ALSGL*-*SB* observation technique, the error of the AE signal estimation using the *ALSGL*-*SB* observation approach, the nonlinear function of the output state for the AE signal in normal conditions using the *ALSGL*-*SB* technique, the uncertainty estimation using the SB observer, the backstepping parameters and the output of the state estimation for AE signals using the *ALSGL*-*SB* technique, respectively. Moreover, the error of AE signal estimation using the *ALSGL*-*SB* observation approach was calculated using the following equation:

(27) $$e_{ALSGL\text{-}SB}\left(k\right)=\Phi_{i}\left(k\right)-H_{ALSGL\text{-}SB}\left(k\right)$$

The uncertainty estimation using the SB observer can be introduced by the following definition:

(28) $$\varphi_{SB}\left(k+1\right)=\varphi_{SB}\left(k\right)+Ζ\left(k\right)+\Upsilon_{i}\psi_{SB}\Phi_{i}\left(k\right)$$

To improve the effect of uncertain estimation accuracy, the combination of *ALSGL*-*SB* algorithm and an integral term, referred to as *ALSGL*-*SBI*, is recommended and introduced by the following equations:

(29) $$\{\begin{array}{l} \Psi_{ALSGL\text{-}SBI}\left(k+1\right)=[\chi_{\mathrm{ALSGL}}\Psi_{ALSGL\text{-}SBI}\left(k\right)\\ +\Upsilon_{H}(H_{ALSGL\text{-}SBI}\left(k\right)+Ζ\left(k\right)]+e_{ALSGL\text{-}SBI}\left(k\right)\\ \;\;\;\;\;+\varphi_{SBI}\left(k\right)+\Upsilon_{i}\psi_{SB}\Phi_{i}\left(k\right)\\ H_{ALSGL\text{-}SBI}\left(k\right)={\left(\Upsilon_{o}\right)}^{T}\left(\omega_{n}\right)\chi_{\mathrm{ALSGL}}{}^{-1}\Psi_{ALSGL\text{-}SBI}\left(k\right) \end{array}$$

where $\Psi_{ALSGL\text{-}SBI}\left(k\right),e_{ALSGL\text{-}SBI}\left(k\right),\varphi_{SBI}\left(k\right)$ and $H_{ALSGL\text{-}SBI}\left(k\right)$ are the state estimation of AE signals using the *ALSGL*-*SBI* observation technique, the error of AE signal estimation using the *ALSGL*-*SBI* observation approach, the uncertainty estimation using the SBI observer and the output of the state estimation for AE signals using the *ALSGL*-*SBI* technique, respectively. The error of AE signal estimation using the *ALSGL*-*SBI* observer was considered using the following equation:

(30) $$e_{ALSGL\text{-}SBI}\left(k\right)=\Phi_{i}\left(k\right)-H_{ALSGL\text{-}SBI}\left(k\right)$$

The uncertainty estimation using the SBI observer can be introduced via the following definition:

(31) $$\varphi_{SBI}\left(k+1\right)=\varphi_{SBI}\left(k\right)+Ζ\left(k\right)+\psi_{SB}e_{ALSGL\text{-}SBI}\left(k\right)+\Upsilon_{i}\psi_{SBI}\Phi_{i}\left(k\right)$$

The *ALSGL*-*SBI* observer faces issues of nonlinear AE signal estimation. To improve the performance of estimation accuracy for AE signals, the combination of *ALSGL*-*SBI* and support vector regression (SVR), referred to as *ALSGL*-*SBIS*, is recommended. *ALSGL*-*SBIS* is introduced by the following definition:

(32) $$\{\begin{array}{l} \Psi_{ALSGL-SBIS}\left(k+1\right)=[\chi_{\mathrm{ALSGL}}\Psi_{ALSGL-SBIS}\left(k\right)\\ +\Upsilon_{H}({Η}_{ALSGL-SBIS}\left(k\right)+Ζ\left(k\right)+{Η}_{SVR}\left(k\right)]+e_{ALSGL-SBIS}\left(k\right)\\ \;\;\;\;\;\;+\varphi_{SBIS}\left(k\right)+\Upsilon_{i}\psi_{SB}\Phi_{i}\left(k\right)\\ {Η}_{ALSGL-SBIS}\left(k\right)={\left(\Upsilon_{o}\right)}^{T}\left(\omega_{n}\right)\chi_{\mathrm{ALSGL}}{}^{-1}\Psi_{ALSGL-SBIS}\left(k\right) \end{array}$$

where $\Psi_{ALSGL\text{-}SBIS}\left(k\right),e_{ALSGL\text{-}SBIS}\left(k\right),\varphi_{SBIS}\left(k\right),$ $H_{SVR}\left(k\right)$ and $H_{ALSGL\text{-}SBIS}\left(k\right)$ are the state estimation of AE signals using the *ALSGL*-*SBIS* observation technique, the error of AE signal estimation using the *ALSGL*-*SBIS* observation approach, the uncertainty estimation using the *SBIS* observer, the AE signal estimation using SVR as calculated by Equation (7) and the output of the state estimation for AE signals using the *ALSGL*-*SBIS* technique, respectively. In addition, the error of AE signal estimation using the *ALSGL*-*SBIS* observer was considered using the following equation:

(33) $$e_{ALSGL\text{-}SBIS}\left(k\right)=\Phi_{i}\left(k\right)-H_{ALSGL\text{-}SBIS}\left(k\right)$$

The uncertainty estimation using the *SBIS* observer can be introduced via the following definition:

(34) $$\varphi_{SBIS}\left(k+1\right)=\varphi_{SBIS}\left(k\right)+Ζ\left(k\right)+\psi_{SB}e_{ALSGL\text{-}SBIS}\left(k\right)+\Upsilon_{i}\psi_{SBIS}\Phi_{i}\left(k\right)+H_{SVR}\left(k\right)$$

To improving the accuracy and reduce the effects of uncertainty estimation, the combination of the *ALSGL*-*SBIS* and TS-fuzzy logic, referred to as the proposed digital twin (PDT), is suggested. The PDT is introduced by the following equations:

(35) $$\{\begin{array}{l} \Psi_{PDT}\left(k+1\right)=[\chi_{\mathrm{ALSGL}}\Psi_{PDT}\left(k\right)\\ +\Upsilon_{H}(H_{PDT}\left(k\right)+Ζ\left(k\right)+H_{SVR}\left(k\right)]+e_{PDT}\left(k\right)\\ \;\;\;\;\;+\varphi_{PDT}\left(k\right)+\Upsilon_{i}\psi_{SB}\Phi_{i}\left(k\right)\\ H_{PDT}\left(k\right)={\left(\Upsilon_{o}\right)}^{T}\left(\omega_{n}\right)\chi_{\mathrm{ALSGL}}{}^{-1}\Psi_{PDT}\left(k\right) \end{array}$$

where $\Psi_{PDT}\left(k\right),e_{PDT}\left(k\right),\varphi_{PDT}\left(k\right),H_{f}\left(k\right),$ and $H_{PDT}\left(k\right)$ are the state estimation of AE signals using the PDT approach, the error of AE signal estimation using the PDT approach, the uncertainty estimation using the PDT approach, the AE signal estimation using the fuzzy technique and the output of the state estimation for AE signals using the PDT technique, respectively. In addition, the error of AE signal estimation using the PDT approach was considered using the following equation:

(36) $$e_{PDT}\left(k\right)=\Phi_{i}\left(k\right)-H_{PDT}\left(k\right)$$

Moreover, the uncertainty estimation using the PDT approach can be introduced by the following definition:

(37) $$\varphi_{PDT}\left(k+1\right)=\varphi_{PDT}\left(k\right)+Ζ\left(k\right)+\psi_{SB}e_{ALSGL\text{-}SBIS}\left(k\right)+\Upsilon_{i}\psi_{SBIS}\Phi_{i}\left(k\right)+H_{SVR}\left(k\right)+H_{F}\left(k\right)$$

The TS-fuzzy approach can be defined as follows:

(38) $$If\;H_{o}\left(k\right)\;is\;\Gamma \;Then\;H_{F}\left(k+1\right)=H_{F}\left(k\right)+\alpha_{f}e_{f}\left(k\right)$$

where $H_{o}\left(k\right),\Gamma ,e_{F}\left(k\right)\;\mathrm{and}$ $\alpha_{f}$ are the output of the signal estimation using the TS-fuzzy approach, the condition’s level, the error of signal estimation based on the fuzzy approach and a coefficient, respectively. The AE signal estimation using the fuzzy technique, $H_{f}\left(k\right),$ can be introduced by the following definition:

(39) $$H_{F}\left(k+1\right)=\frac{\sum H_{F}\left(k\right)\prod \mu \left(e_{f}\left(k\right)\right)}{\sum \prod \mu \left(e_{f}\left(k\right)\right)}$$

where $\mu \left(e_{f}\left(k\right)\right)$ is the membership of the error. Moreover, the error of the signal estimation using the T-S fuzzy approach is given by the following equation:

(40) $$e_{F}\left(k\right)=\Phi_{i}\left(k\right)-H_{F}\left(k\right)$$

**Figure 6:**
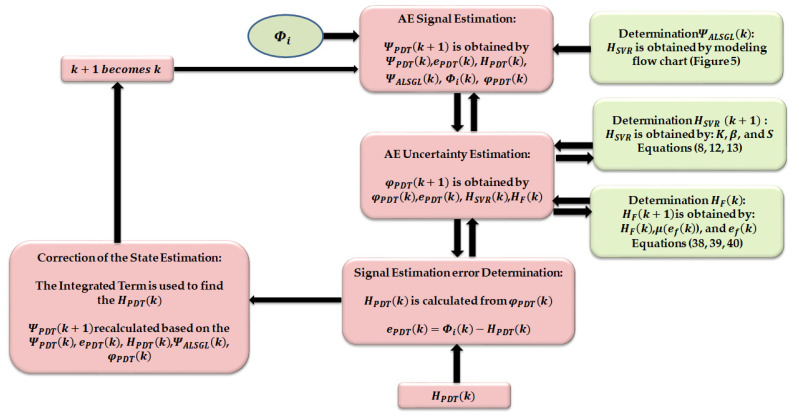
Flow chart typical of AE signal estimation using proposed digital twin.

Thus, based on these definitions, the PDT was designed based on a combination of data-driven AE signal modeling and AE signal estimation to estimate the unknown AE signals. Figure 6 illustrates the flowchart of the AE signal estimation using the proposed digital twin. In the next part, the generation of the AE residual signal is presented.

### 3.2. Acoustic Emission Residual Signal Generation

After modeling and estimation of the unknown AE signal using the PDT, the residual signal, which is the difference between the measurable AE signal and the estimated AE signal, can be calculated using the following equation:

(41) $$R_{PDT}\left(k\right)=\Phi_{i}\left(k\right)-H_{PDT}\left(k\right)$$

### 3.3. Crack Diagnosis Using the Machine Learning Approach

After generating the residual AE signals using the PDT algorithm, the root-mean-square (RMS) feature was extracted from residual signals using the following equation:

(42) $$R_{PDT}{\left(k\right)}_{rms}=\sqrt{\frac{1}{K}\sum_{i=1}^{K}{\left(R_{PDT}\left(k\right)\right)}^{2}}$$

where $R_{PDT}{\left(k\right)}_{rms}$ and *K* are the resampled RMS value for the AE residual signals calculated using the PDT algorithm and the number of windows, respectively.

The number of samples for the healthy and each unhealthy condition was 120,000. The residual signal was divided into 100 windows for each state. It means that we obtained the RMS feature for each window (every 1200 samples). So, we had 100 new RMS samples for each state. On the other hand, based on Table 3, we had eight different conditions. Each faulty conditions had four different speeds (300 RPM, 400 RPM, 450 RPM and 500 RPM) and two different crack sizes (3 mm and 6 mm). Thus, in each condition (for example inner fault), we had eight parts (four different speeds multiplied by two different crack sizes). Thus, we had 800 new RMS samples for each type of fault. Moreover, the support vector machine (SVM) was selected for bearing crack diagnosis [30]. The SVM is known as a robust machine learning algorithm that is impervious to the curse of the dimensionality problem. One of the main advantages of the SVM is that it can be proficiently applied for the classification of both linear and nonlinear separable types of datasets, which is possible due to the availability of different types of kernels, such as linear, polynomial and radial basis function kernels [30]. In this work, 75% of the resampled RMS residual signal was used for training and 25% was used for testing. Thus, 0.75 × 800 resampled RMS signal was used for training and 0.25 × 800 resampled RMS signal was used for testing. Table 3 presents the details of the training and testing data for crack detection and diagnosis.

Algorithm 1 illustrates the steps for designing the proposed digital twin, machine learning and backstepping for leak detection and localization in a chemical plant.
**Algorithm 1** Proposed strict-feedback backstepping digital twin and machine learning solution algorithm for bearing fault diagnosis.
**Step 1.1: Acoustic Emission (AE) Signal Modeling**1:Acoustic Emission (AE) signal modeling using the AR technique; Equation (1)
**Detail**1.1Calculate $e_{A}\left(k\right)\leftarrow H_{A}\left(k\right)-H_{A}\left(k-1\right)$, Equation (2)1.2Compute $\Psi_{A}\left(k+1\right)\leftarrow \left[\sigma_{\Psi }\Psi_{A}\left(k\right)+\sigma_{i}\Phi_{i}\left(k\right)\right]+e_{A}\left(k\right)+\varphi \left(k\right)$, Equation (1)1.3Compute $H_{A}\left(k\right)={\left(\sigma_{o}\right)}^{T}\Psi_{A}\left(k\right)$, (1)2:Improving the robustness of the AR technique for AE signal modeling using a Laguerre filter (AL); Equation (3)
**Detail**2.1Calculate $e_{AL}\left(k\right)\leftarrow H_{AL}\left(k\right)-H_{AL}\left(k-1\right)$, (4)2.2Compute $\Psi_{AL}\left(k+1\right)\leftarrow \left[\sigma_{\Psi }\Psi_{AL}\left(k\right)+\sigma_{i}\Phi_{i}\left(k\right)+\sigma_{H}H_{AL}\left(k\right)\right]+e_{AL}\left(k\right)+\varphi \left(k\right),$ (3)2.3Compute $H_{AL}\left(k\right)={\left(\sigma_{o}\right)}^{T}\Psi_{AL}\left(k\right)$. (3)3:Reducing the effects of complexity and nonlinearity of the AL technique for AE signal modeling using support vector regression, ALS; Equation (5)
**Detail**3.1Compute $K\left(\phi_{i},\phi \right)\leftarrow e^{\left(-\frac{1}{2\varepsilon^{2}}||\phi_{i}-\phi |{|}^{2}\right)},$ (8)3.2Compute $S=\left\{i|0<\varrho_{i}{}^{+}+\varrho_{i}{}^{-}<\eta \right\},$ (13)3.3Calculate $\beta \leftarrow \frac{1}{\left|S\right|}\underset{s\in S}{\sum }\left[\begin{array}{l} H_{s}-\underset{i\in S}{\sum }\left(\varrho_{i}{}^{+}-\varrho_{i}{}^{-}\right)\times \\ K\left(\phi_{i},\phi \right)-\rho \\ \times sgn\left(\varrho_{i}{}^{+}-\varrho_{i}{}^{-}\right) \end{array}\right],$ (12)3.4Compute $H_{SVR}\leftarrow \underset{i}{\sum }\left(\varrho_{i}{}^{+}-\varrho_{i}{}^{-}\right)K\left(\phi_{i},\phi \right)+\beta ,$ (7)3.5Calculate $e_{ALS}\left(k\right)\leftarrow H_{ALS}\left(k\right)-H_{ALS}\left(k-1\right)$, (6)3.6Compute $H_{ALS}\left(k\right)\leftarrow {\left(\sigma_{o}\right)}^{T}\Psi_{ALS}\left(k\right),$ (5)3.7Compute $\Psi_{ALS}\left(k+1\right)\leftarrow \left[\sigma_{\Psi }\Psi_{ALS}\left(k\right)+\sigma_{i}\Phi_{i}\left(k\right)+\sigma_{H}H_{ALS}\left(k\right)+\sigma_{SVR}H_{SVR}\left(k\right)\right]+e_{ALS}\left(k\right)+\varphi \left(k\right).$ (5)4:Acoustic emission (AE) signal modeling using the GPR technique; Equation (15)
**Detail**4.1Compute $W\leftarrow diag{\left(T\right)}^{2},$ (18)4.2Calculate $\chi_{G}\leftarrow \gamma^{2}e^{\left(-0.5\Psi_{G}{}^{T}W^{-1}\Psi_{G}\right)}+\vartheta ,$ (17)4.3Compute $e_{G}\left(k\right)\leftarrow H_{G}\left(k\right)-H_{G}\left(k-1\right)$ (16)4.4Calculate $\Psi_{G}\left(k+1\right)\leftarrow \left[\chi_{G}\Psi_{G}\left(k\right)+\Upsilon_{i}\Phi_{i}\left(k\right)\right]+e_{G}\left(k\right)+\varphi \left(k\right),$ (15)4.5Compute $H_{G}\left(k\right)\leftarrow {\left(\Upsilon_{o}\right)}^{T}\left(\omega_{n}\right)\chi_{G}{}^{-1}\Psi_{G}\left(k\right).$ (15)5:Improving the robustness of the GPR technique for AE signal modeling using a Laguerre filter (GL); Equation (19)
**Detail**5.1Compute $\chi_{GL}\leftarrow \gamma^{2}e^{\left(-0.5\Psi_{GL}{}^{T}W^{-1}\Psi_{GL}\right)}+\vartheta ,$ (21)5.2Compute $e_{GL}\left(k\right)\leftarrow H_{GL}\left(k\right)-H_{GL}\left(k-1\right),$ (20)5.3Calculate $\Psi_{GL}\left(k+1\right)\leftarrow \left[\chi_{GL}\Psi_{GL}\left(k\right)+\Upsilon_{i}\Phi_{i}\left(k\right)+\Upsilon_{H}H_{GL}\left(k\right)\right]+e_{GL}\left(k\right)+\varphi \left(k\right),$ (19)5.4Compute $H_{GL}\left(k\right)\leftarrow {\left(\Upsilon_{o}\right)}^{T}\left(\omega_{n}\right)\chi_{GL}{}^{-1}\Psi_{GL}\left(k\right).$ (19)6:Increasing the accuracy and reliability of AE signal modeling using Gaussian process regression and a Laguerre filter with the ALS approach, ALSGL; Equation (23)
**Detail**6.1Compute $\Psi_{ALSGL}\left(k+1\right)\leftarrow \Psi_{ALS}\left(k\right)+\Psi_{GL}\left(k\right),$ (22)6.2Compute $\Psi_{ALSGL}\left(k+1\right)\leftarrow \left[\chi_{\mathrm{ALSGL}}\Psi_{ALSGL}\left(k\right)+\Upsilon_{i}\Phi_{i}\left(k\right)+\Upsilon_{H}H_{ALSGL}\left(k\right)\right]+e_{ALSGL}\left(k\right)+\varphi \left(k\right),$ (23)6.3Calculate $H_{ALSGL}\left(k\right)\leftarrow H_{ALS}\left(k\right)+H_{GL}\left(k\right).$ (22)6.4Compute $H_{ALSGL}\left(k\right)\leftarrow {\left(\Upsilon_{o}\right)}^{T}\left(\omega_{n}\right)\chi_{\mathrm{ALSGL}}{}^{-1}\Psi_{ALSGL}\left(k\right).$ (23)6.5Compute $e_{ALSGL}\left(k\right)\leftarrow H_{ALSGL}\left(k\right)-H_{ALSGL}\left(k-1\right).$ (24)
**Step 1.2: Acoustic Emission (AE) Signal Estimation Using the Proposed Digital Twin**7:Reducing the effects of uncertainties in AE signal modeling using ALS-GL and the proposed strict-feedback observer, *ALSGL*-*SB*; Equations (26) and (28).
**Detail**7.1Compute $\Psi_{ALSGL\text{-}SB}\left(k+1\right)\leftarrow \left[\chi_{\mathrm{ALSGL}}\Psi_{ALSGL-SB}\left(k\right)+\Upsilon_{H}(H_{ALSGL-SB}\left(k\right)+Ζ\left(k\right)\right]+e_{ALSGL\text{-}SB}\left(k\right)+\varphi_{SB}\left(k\right)+\Upsilon_{i}\psi_{SB}\Phi_{i}\left(k\right),$ (26)7.2Calculate $H_{ALSGL\text{-}SB}\left(k\right)\leftarrow {\left(\Upsilon_{o}\right)}^{T}\left(\omega_{n}\right)\chi_{\mathrm{ALSGL}}{}^{-1}\Psi_{ALSGL\text{-}SB}\left(k\right).$ (26)7.3Compute $e_{ALSGL\text{-}SB}\left(k\right)\leftarrow \Phi_{i}\left(k\right)-H_{ALSGL\text{-}SB}\left(k\right),\;$(27)7.4Calculate $\varphi_{SB}\left(k+1\right)\leftarrow \varphi_{SB}\left(k\right)+Ζ\left(k\right)+\Upsilon_{i}\psi_{SB}\Phi_{i}\left(k\right).$ (28)8:Improving the effects of uncertain estimation accuracy for AE signals using the *ALSGL*-*SB* algorithm and integral term, *ALSGL*-*SBI*; Equations (29) and (31).
**Detail**8.1Compute $\Psi_{ALSGL\text{-}SBI}\left(k+1\right)\leftarrow \left[\chi_{\mathrm{ALSGL}}\Psi_{ALSGL-SBI}\left(k\right)+\Upsilon_{H}(H_{ALSGL-SBI}\left(k\right)+Ζ\left(k\right)\right]+e_{ALSGL\text{-}SBI}\left(k\right)+\varphi_{SBI}\left(k\right)+\Upsilon_{i}\psi_{SB}\Phi_{i}\left(k\right),$ (29)8.2Calculate $H_{ALSGL\text{-}SBI}\left(k\right)\leftarrow {\left(\Upsilon_{o}\right)}^{T}\left(\omega_{n}\right)\chi_{\mathrm{ALSGL}}{}^{-1}\Psi_{ALSGL\text{-}SBI}\left(k\right).$ (29)8.3Solve $e_{ALSGL\text{-}SBI}\left(k\right)\leftarrow \Phi_{i}\left(k\right)-H_{ALSGL\text{-}SBI}\left(k\right),$ (30)8.4Compute $\varphi_{SBI}\left(k+1\right)\leftarrow \varphi_{SBI}\left(k\right)+Ζ\left(k\right)+\psi_{SB}e_{ALSGL\text{-}SBI}\left(k\right)+\Upsilon_{i}\psi_{SBI}\Phi_{i}\left(k\right).$ (31)9:Improving the performance of estimation accuracy for AE signals using *ALSGL*-*SBI* and support vector regression, *ALSGL*-*SBIS*; Equations (32) and (34).
**Detail**9.1Calculate $\Psi_{ALSGL\text{-}SBIS}\left(k+1\right)\leftarrow \left[\chi_{\mathrm{ALSGL}}\Psi_{ALSGL-SBIS}\left(k\right)+\Upsilon_{H}(H_{ALSGL-SBIS}\left(k\right)+Ζ\left(k\right)+H_{SVR}\left(k\right)\right]+e_{ALSGL\text{-}SBIS}\left(k\right)+\varphi_{SBIS}\left(k\right)+\Upsilon_{i}\psi_{SB}\Phi_{i}\left(k\right),$ (32)9.2Solve $H_{ALSGL\text{-}SBIS}\left(k\right)\leftarrow {\left(\Upsilon_{o}\right)}^{T}\left(\omega_{n}\right)\chi_{\mathrm{ALSGL}}{}^{-1}\Psi_{ALSGL\text{-}SBIS}\left(k\right),$ (32)9.3Compute $e_{ALSGL\text{-}SBIS}\left(k\right)\leftarrow \Phi_{i}\left(k\right)-H_{ALSGL\text{-}SBIS}\left(k\right),$ (33)9.4Calculate $\varphi_{SBIS}\left(k+1\right)\leftarrow \varphi_{SBIS}\left(k\right)+Ζ\left(k\right)+\psi_{SB}e_{ALSGL\text{-}SBIS}\left(k\right)+\Upsilon_{i}\psi_{SBIS}\Phi_{i}\left(k\right)+H_{SVR}\left(k\right).$ (34)10:Improving the accuracy and reducing the effects of uncertainty estimation for AE signals using *ALSGL*-*SBIS* and TS-fuzzy logic, referred to as the proposed digital twin (PDT); Equations (35) and (37)
**Detail**10.1Solve $\Psi_{PDT}\left(k+1\right)=\left[\chi_{\mathrm{ALSGL}}\Psi_{PDT}\left(k\right)+\Upsilon_{H}(H_{PDT}\left(k\right)+Ζ\left(k\right)+H_{SVR}\left(k\right)\right]+e_{PDT}\left(k\right)+\varphi_{PDT}\left(k\right)+\Upsilon_{i}\psi_{SB}\Phi_{i}\left(k\right),$ (35)10.2Compute $H_{PDT}\left(k\right)={\left(\Upsilon_{o}\right)}^{T}\left(\omega_{n}\right)\chi_{\mathrm{ALSGL}}{}^{-1}\Psi_{PDT}\left(k\right).$ (35)10.3Calculate $e_{PDT}\left(k\right)=\Phi_{i}\left(k\right)-H_{PDT}\left(k\right),$ (36)10.4Solve $\varphi_{PDT}\left(k+1\right)=\varphi_{PDT}\left(k\right)+Ζ\left(k\right)+\psi_{SB}e_{PDT}\left(k\right)+\Upsilon_{i}\psi_{PDT}\Phi_{i}\left(k\right)+H_{SVR}\left(k\right)+H_{F}\left(k\right).$ (37)
**Step 2: Acoustic Emission Residual Signal Generation**11:Generating AE residual signals using the difference between the original AE signals and PDT-based estimated AE signals; Equation (41)
**Detail**11.1Compute $R_{PDT}\left(k\right)=\Phi_{i}\left(k\right)-H_{PDT}\left(k\right).$ (41)
**Step 3: Crack Diagnosis Using Machine Learning Approach**12.1:RMS feature extraction from the AE residual signal; Equation (42)12.2Crack detection and diagnosis using SVM [30].

## 4. Experimental Results

**Figure 7:**
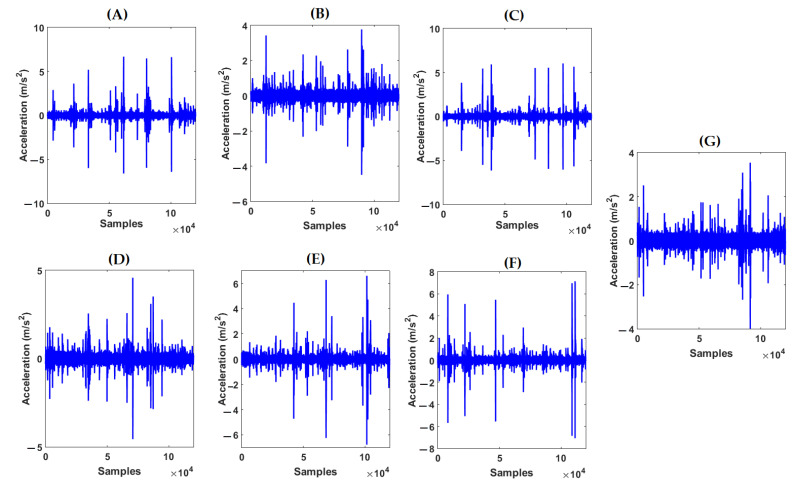
Acoustic emission original abnormal signals: (**A**) ball condition, (**B**) outer condition, (**C**) inner condition, (**D**) inner–outer condition, (**E**) inner–ball condition, (**F**) outer–ball condition and (**G**) inner–outer–ball condition.

**Figure 8:**
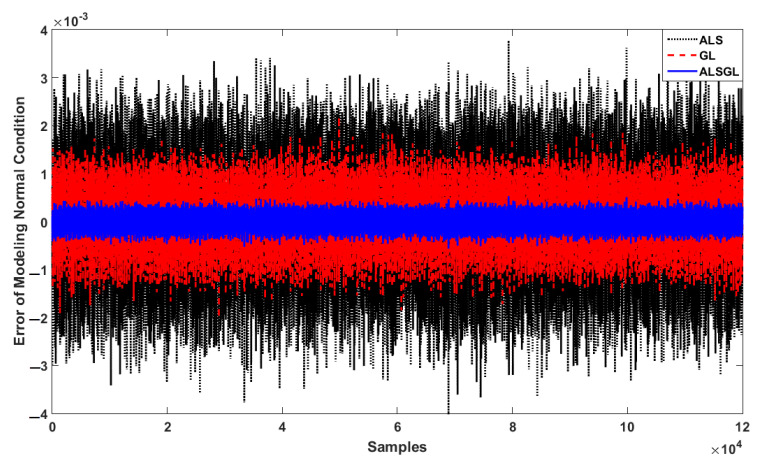
Error of AE signal modeling using ALS, GL and proposed ALSGL approaches.

The Ulsan Industrial Artificial Intelligence (UIAI) Lab acoustic emission (AE) dataset was used to test the power of PDT for crack diagnosis in the bearing. Figure 7 shows the AE bearing signals for all abnormal conditions. Figure 8 illustrates the error of AE signal modeling in healthy conditions based on the ALS technique, GL method and proposed ALSGL approach. Regarding this figure, the error of the proposed ALSGL was less than that of the other two methods. The main reason for errors during system (signal) modeling was uncertain conditions.

**Figure 9:**
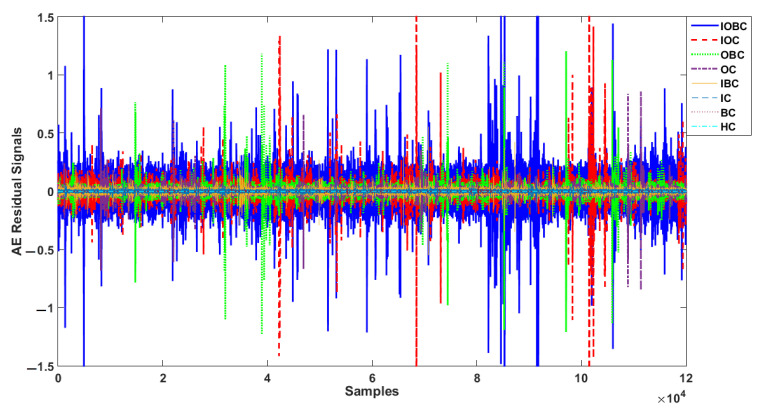
AE residual signals for normal and abnormal conditions using proposed digital twin.

Figure 9 shows the residual signal based on the proposed digital twin (PDT). The visibility of the PDT was perfect for classifying the bearing conditions. Based on this figure, the residual signal had 120,000 samples and had 8 different conditions, including HC, BC, IC, OC, IBC, OBC, IOC and IOBC. The minimum residual signal was for the healthy condition. Because the signal was modeled for this condition and we had the best estimation accuracy, the residual signal was minimum in this condition.

**Figure 10:**
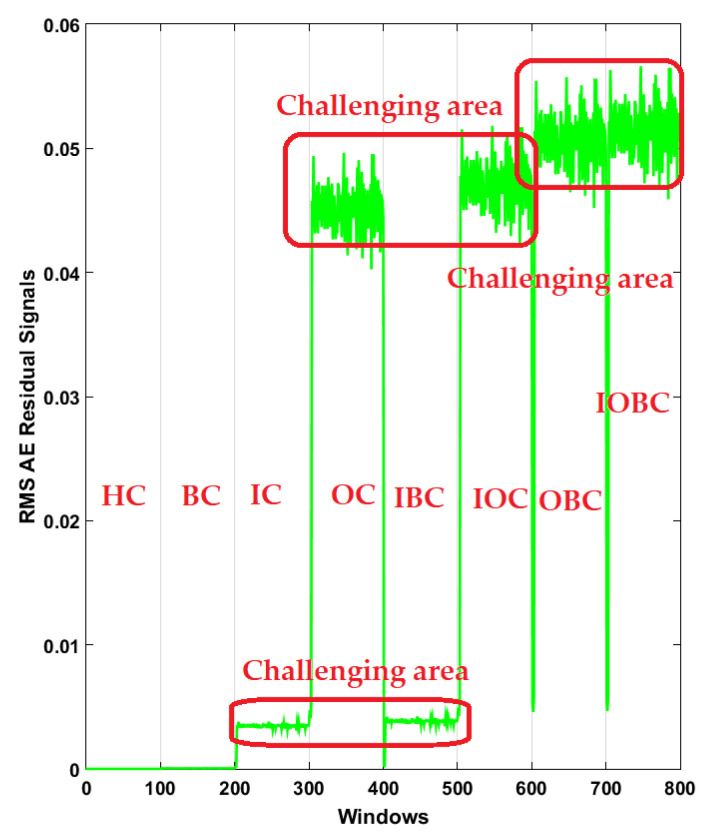
RMS of the resampled AE residual signals for crack type diagnosis in normal and abnormal conditions using *ALSGL*-*SB* method.

**Figure 11:**
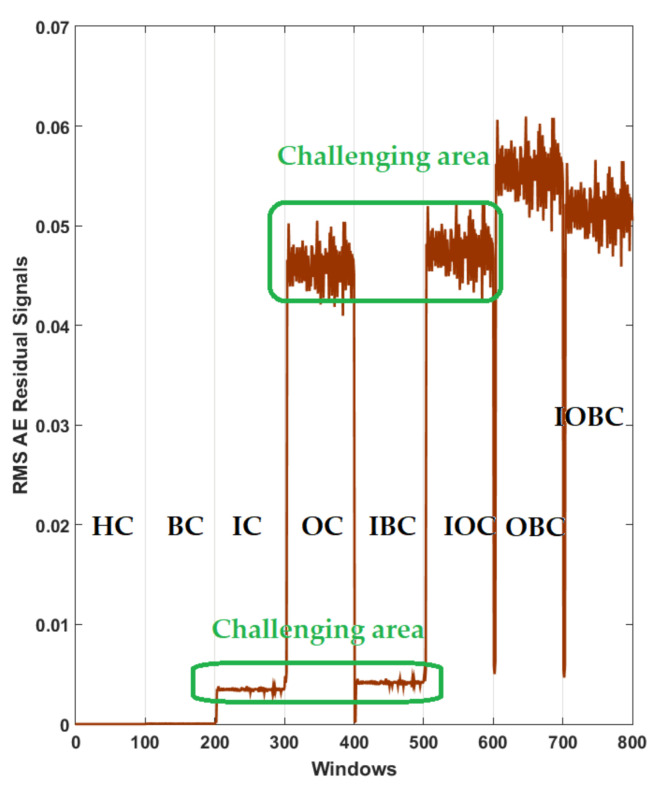
RMS of the resampled AE residual signals for crack type diagnosis in normal and abnormal conditions using the *ALSGL*-*SBI* technique.

**Figure 12:**
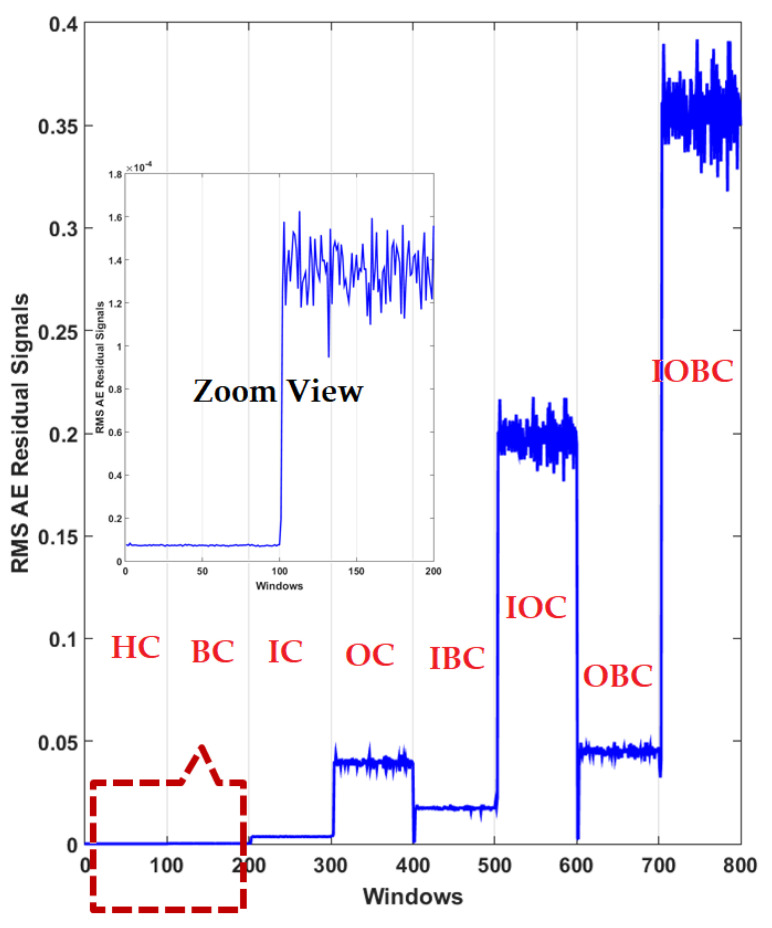
RMS of the resampled AE residual signals for crack type diagnosis in normal and abnormal conditions using the proposed digital twin (PDT).

Additionally, to test the power of AE signal estimation for crack type diagnosis and have a clear visibility, the RMS of the resampled AE residual signals for normal and abnormal states based on a classical strict-feedback backstepping (*ALSGL*-*SB*) observer, the combination of the *ALSGL*-*SB* algorithm and integral term (*ALSGL*-*SBI*) and PDT approaches are illustrated in Figure 10, Figure 11 and Figure 12, respectively. Figure 10 illustrates the power of AE signal estimation for crack type diagnosis of the RMS of the resampled AE residual signals for normal and abnormal states based on a classical strict-feedback backstepping (*ALSGL*-*SB*) observer. Based on this figure, the level of the signal in some conditions of bearing, including IC, IBC, OC, IOC, OBC and IOBC, had overlapping. These overlaps in different cases reduced the accuracy of the classification. 

Furthermore, Figure 11 shows the power of AE signal estimation for crack type diagnosis of the RMS of the resampled AE residual signals for normal and abnormal states based on a combination of the *ALSGL*-*SB* algorithm and integral term (*ALSGL*-*SBI*).

Based on Figure 11, the level of the signal in some conditions of bearing, including IC, IBC, OC and IOC, had overlapping. Compared to the *ALSGL*-*SB* technique, the *ALSGL*-*SBI* method had lower overlapping, which can cause an increase in the accuracy of the classification.

Additionally, Figure 12 shows the power of AE signal estimation for crack type diagnosis of the RMS of the resampled AE residual signals for normal and abnormal states based on the proposed digital twin (PDT). Based on this figure, the overlapping was very low. therefore, the power of separability of the PDT was better than that of the other two methods, that could have caused it to have the highest accuracy for classification.

**Figure 13:**
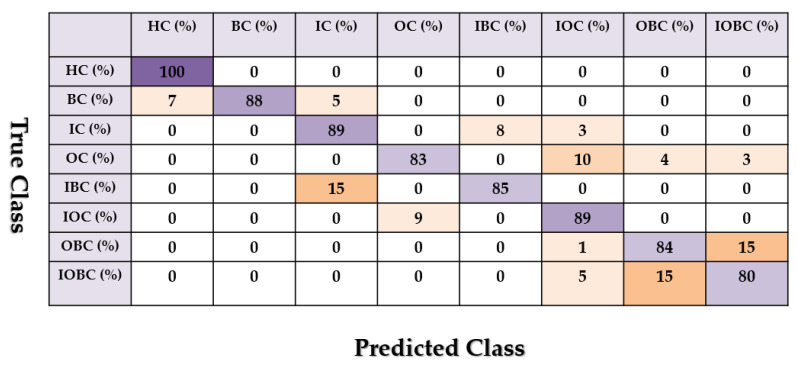
The average of crack type diagnosis using the combination of the *ALSGL*-*SB* and SVM approaches.

**Figure 14:**
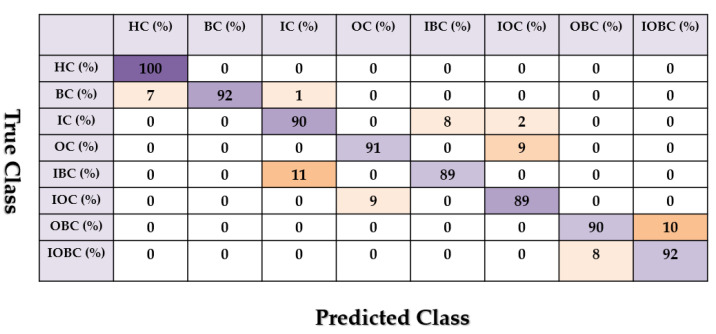
The average of crack type diagnosis using the combination of the *ALSGL*-*SBI* and SVM approaches.

**Figure 15:**
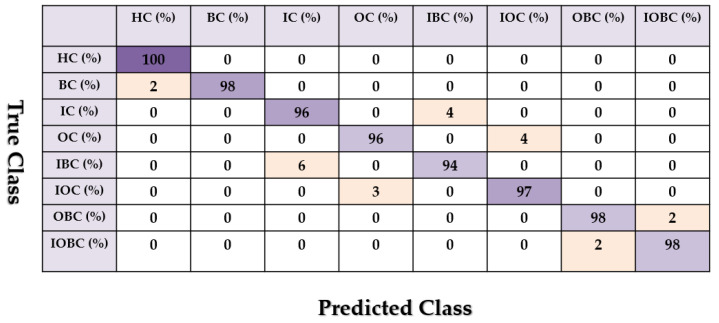
The average of crack type diagnosis using the combination of the PDT and SVM approaches.

Figure 13, Figure 14 and Figure 15 show the confusion matrix for the combined *ALSGL*-*SB* and SVM, combined *ALSGL*-*SBI* and SVM and combined PDT and SVM for crack type diagnosis, respectively.

Based on these figures, the range of misclassification accuracy using the combined PDT and SVM methods was less than that of the other two techniques. Moreover, Table 4 shows the average accuracy of crack diagnosis.

**Figure 16:**
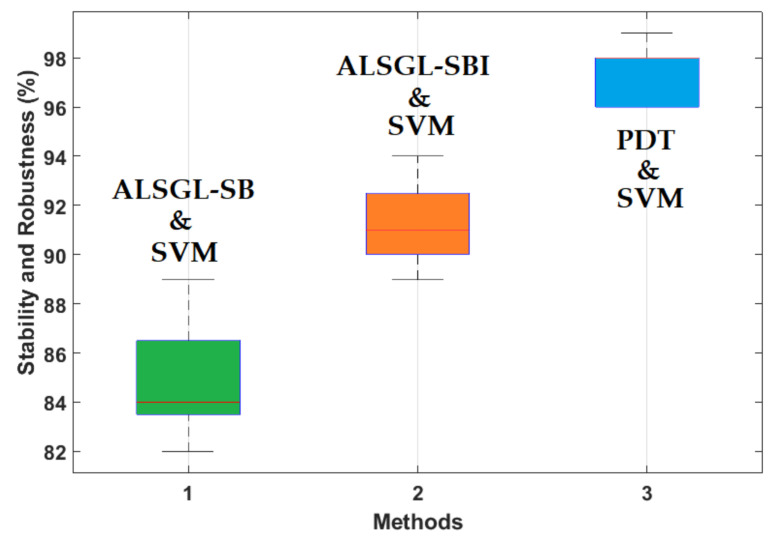
Test of reliability and robustness for crack type diagnosis using the combination of the *ALSGL*-*SB* and SVM approaches, the *ALSGL*-*SBI* and SVM methods and the combination of the PDT and SVM procedures (20 times).

Based on this table, the average accuracy of crack type diagnosis for various crack sizes (3 mm and 6 mm) and motor speeds (300 RPM, 400 RPM, 450 RPM and 500 RPM) were 87.25%, 91.63% and 97.13% for the combined *ALSGL*-*SB* and SVM, *ALSGL*-*SBI* and SVM, and PDT and SVM approaches, respectively. Thus, the combination of PDT and SVM improved the average accuracy for crack type diagnosis by 9.88% compared with the combination of *ALSGL*-*SB* and SVM and by 5.5% compared with the combination of *ALSGL*-*SBI* and SVM. To test the stability and robustness of the fault type diagnosis, the experiment was repeated 20 times with a random selection of samples to form the train and test sets each time [43,44]. Figure 16 shows the robustness of the combined *ALSGL*-*SB* and SVM, *ALSGL*-*SBI* and SVM, and PDT and SVM approaches. Based on this figure, the proposed method was more robust than the other two approaches and the fluctuations were less than two other techniques. 

**Figure 17:**
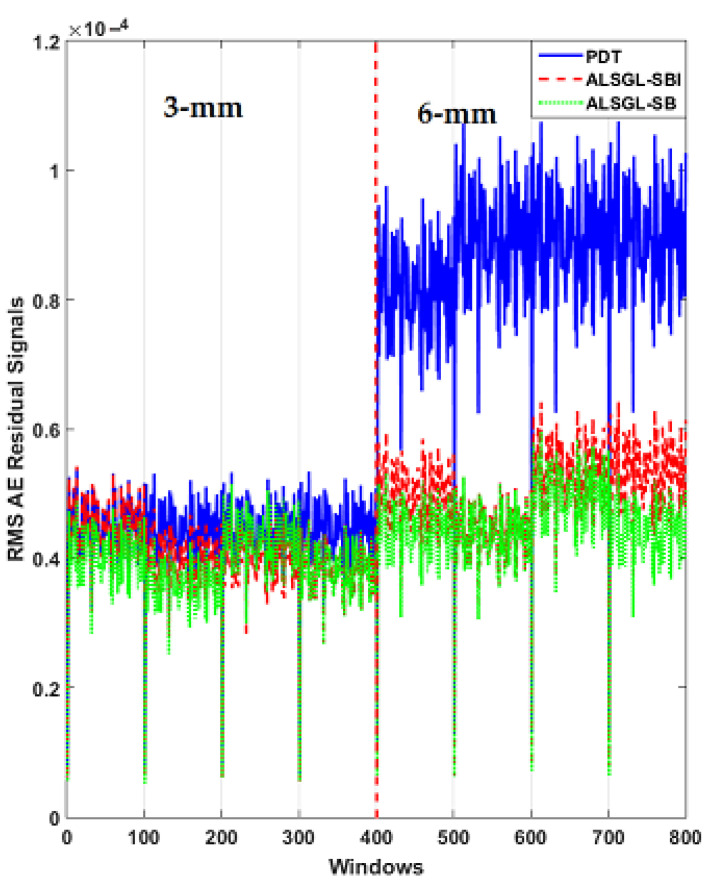
RMS of resampled AE residual signals for crack size (3 mm and 6 mm) diagnosis for ball conditions using *ALSGL*-*SB*, *ALSGL*-*SBI* and PDT methods.

**Figure 18:**
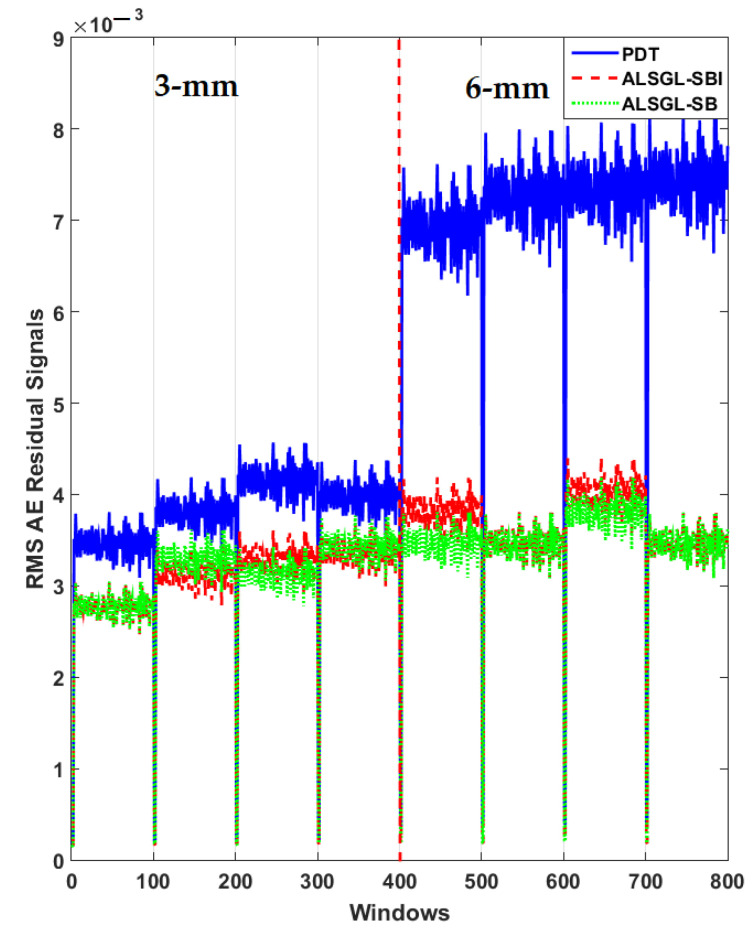
RMS of resampled AE residual signals for crack size (3 mm and 6 mm) diagnosis for inner conditions using *ALSGL*-*SB*, *ALSGL*-*SBI* and PDT methods.

**Figure 19:**
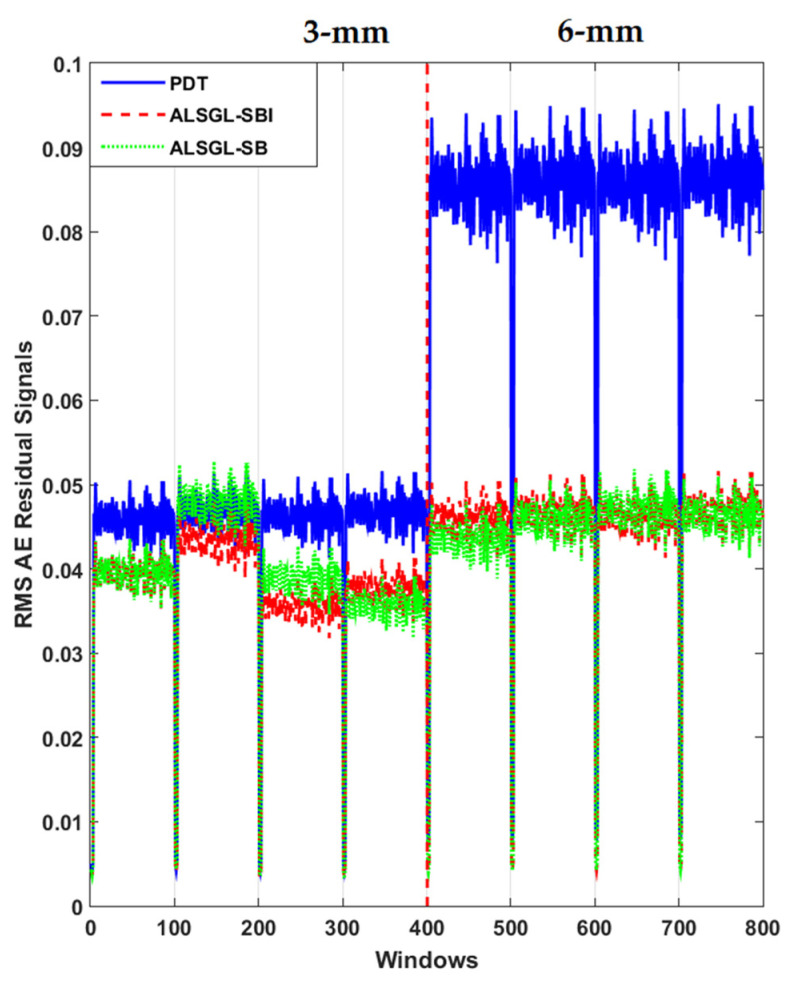
RMS of resampled AE residual signals for crack size (3 mm and 6 mm) diagnosis for outer conditions using *ALSGL*-*SB*, *ALSGL*-*SBI* and PDT methods.

**Figure 20:**
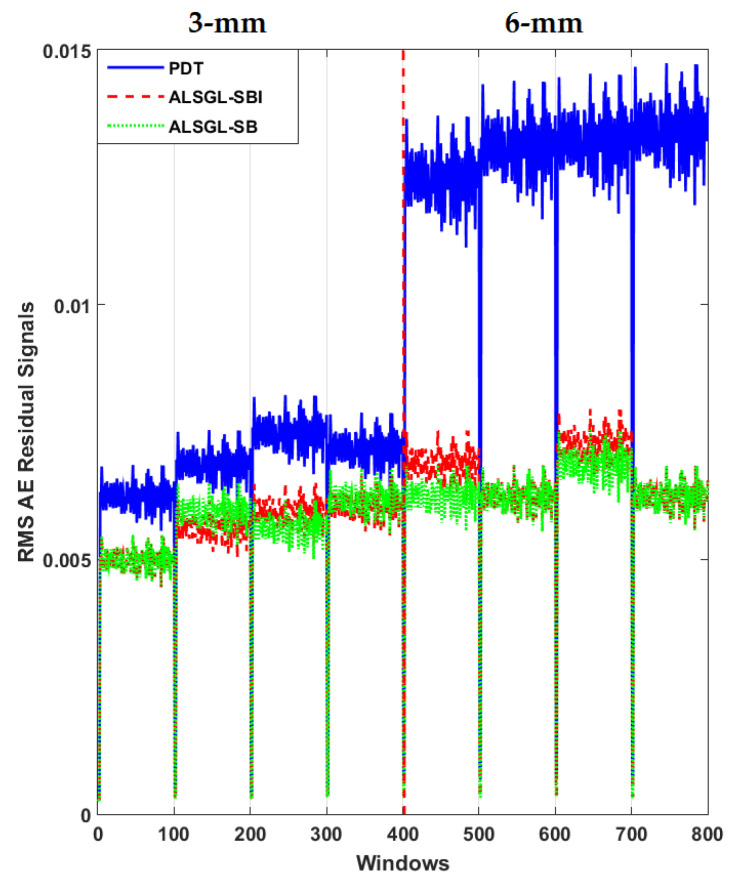
RMS of resampled AE residual signals for crack size (3 mm and 6 mm) diagnosis for inner–ball conditions using *ALSGL*-*SB*, *ALSGL*-*SBI* and PDT methods.

**Figure 21:**
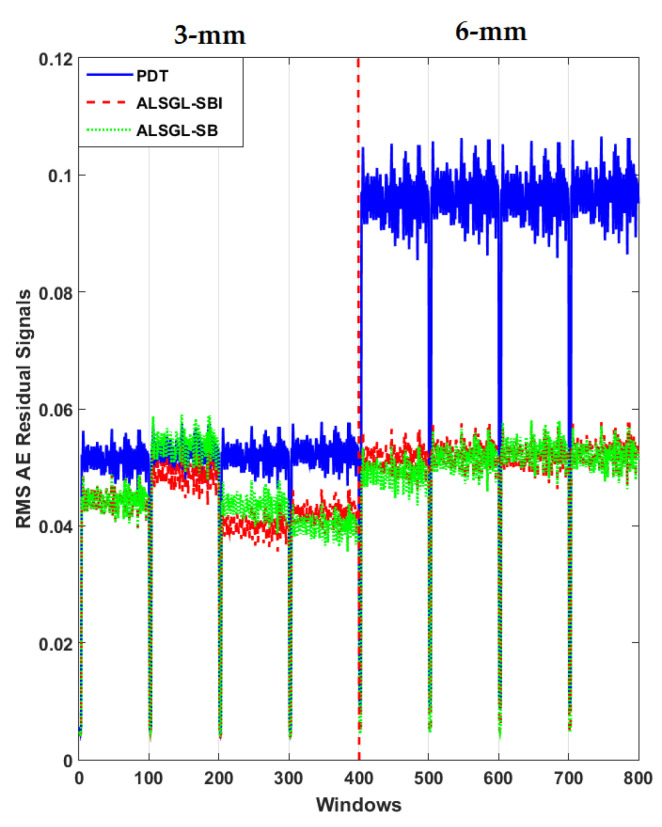
RMS of resampled AE residual signals for crack size (3 mm and 6 mm) diagnosis for outer–ball conditions using *ALSGL*-*SB*, *ALSGL*-*SBI* and PDT methods.

**Figure 22:**
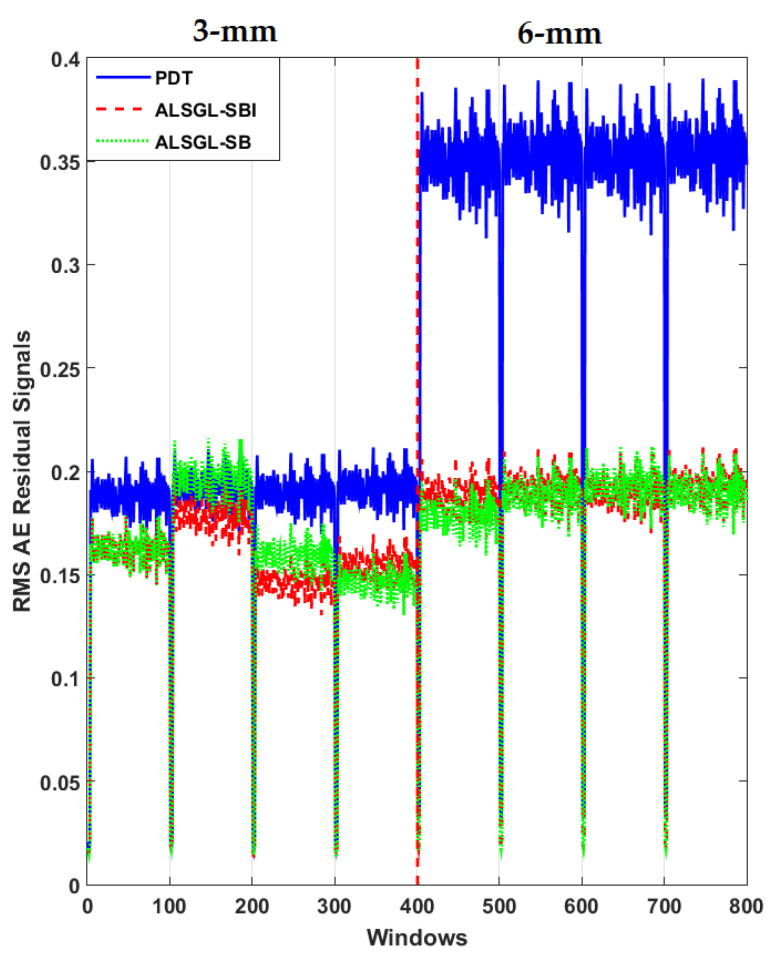
RMS of resampled AE residual signals for crack size (3 mm and 6 mm) diagnosis for inner–outer conditions using *ALSGL*-*SB*, *ALSGL*-*SBI* and PDT methods.

**Figure 23:**
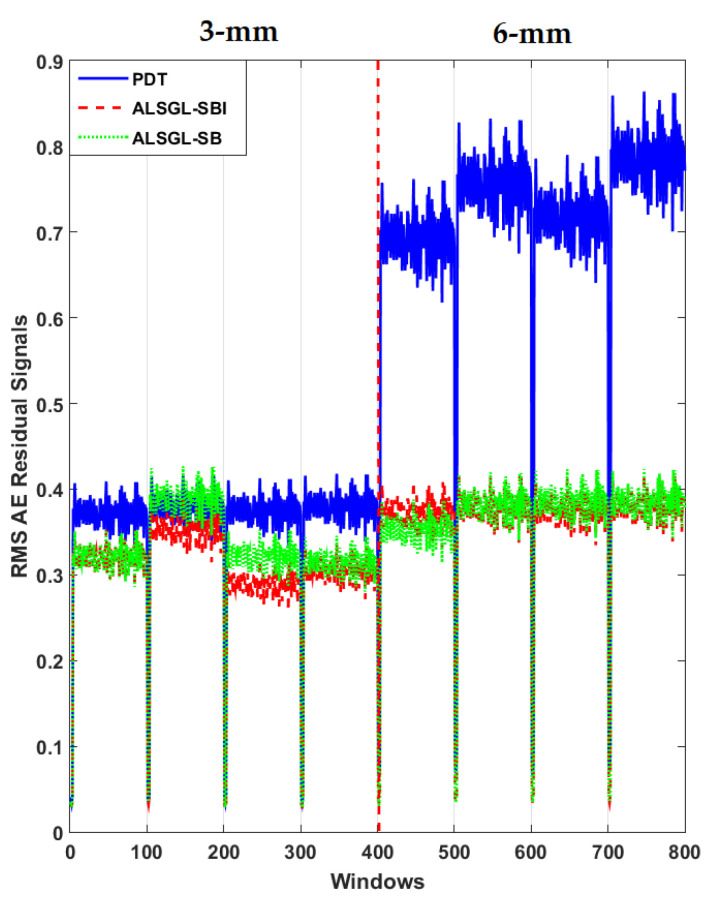
RMS of resampled AE residual signals for crack size (3 mm and 6 mm) diagnosis for inner–outer–ball conditions using *ALSGL*-*SB*, *ALSGL*-*SBI* and PDT methods.

Furthermore, to test the power of AE signal estimation for crack size (3 mm and 6 mm) diagnosis, the RMS of the resampled AE residual signals for normal and abnormal states, including BC, IC, OC, IBC, OBC, IOC and IOBC, based on a classical strict-feedback backstepping (*ALSGL*-*SB*) observer, the combined *ALSGL*-*SB* algorithm and integral term (*ALSGL*-*SBI*) and PDT approaches are illustrated in Figure 17, Figure 18, Figure 19, Figure 20, Figure 21, Figure 22 and Figure 23, respectively.

**Figure 24:**
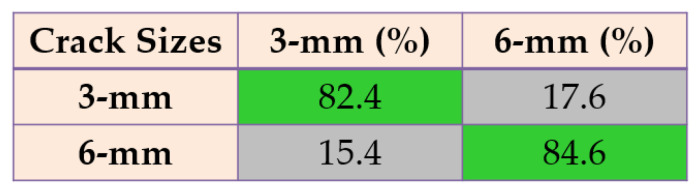
The average of crack size (3 mm and 6 mm) diagnosis using the combination of *ALSGL*-*SB* and SVM approach for all abnormal conditions.

**Figure 25:**
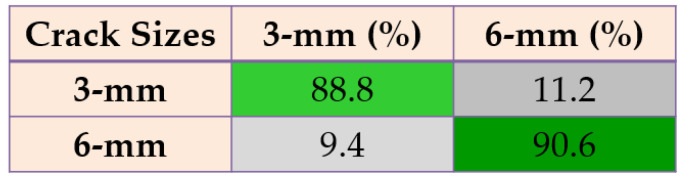
The average of crack size (3 mm and 6 mm) diagnosis using the combination of *ALSGL*-*SBI* and SVM approach for all abnormal conditions.

**Figure 26:**
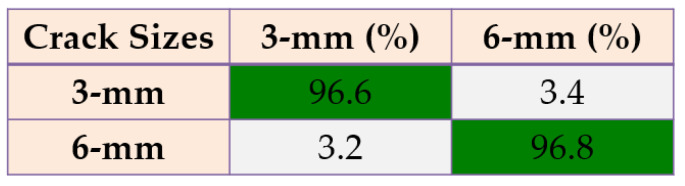
The average of crack size (3 mm and 6 mm) diagnosis using the combination of PDT and SVM approach for all abnormal conditions.

Based on these figures, the power of separability for crack size diagnosis of the PDT was better than that of the other two techniques. Figure 24, Figure 25 and Figure 26 provide the confusion matrices for the combined *ALSGL*-*SB* observer and SVM, *ALSGL*-*SBI* and SVM, and PDT and SVM approaches for crack size diagnosis, respectively. 

Based on these figures, the range of misclassification accuracy using the combined PDT and SVM methods for crack size diagnosis was smaller than that of the other two techniques. Moreover, Table 5 displays the average accuracy of crack size diagnosis for the combined SBO and SVM, *ALSGL*-*SBI* and SVM, and PDT and SVM techniques.

**Figure 27:**
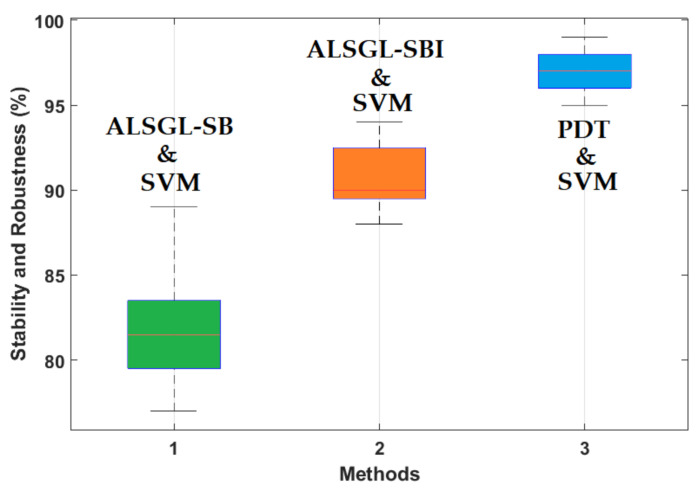
Test of reliability and robustness for crack size (3 mm and 6 mm) diagnosis using the combination of the *ALSGL*-*SB* and SVM approaches, *ALSGL*-*SBI* and SVM methods and the combination of the PDT and SVM procedures (20 times).

Based on this table, the average accuracy for crack size diagnosis for various conditions and motor speeds (300 RPM, 400 RPM, 450 RPM and 500 RPM) were 83.1%, 89.7% and 96.9% for the combined *ALSGL*-*SB* and SVM, *ALSGL*-*SBI* and SVM, and PDT and SVM approaches, respectively. Thus, the combination of PDT and SVM improved the average accuracy for crack size diagnosis by 13.8% compared with the combined *ALSGL*-*SB* and SVM method and by 7.2% compared with the combined *ALSGL*-*SBI* and SVM method. To test the stability and robustness of crack size identification, the experiment was repeated 20 times with a random selection of samples to form the train and test sets each time [43,44]. Figure 27 shows the robustness of the combined *ALSGL*-*SB* and SVM, *ALSGL*-*SBI* and SVM, and PDT and SVM methods. 

Based on this figure, the proposed method was more robust than the other two approaches for crack size diagnosis.

## 5. Conclusions

In this research study, a strict-feedback backstepping digital twin for bearing crack diagnosis was proposed. The proposed feedback backstepping digital twin has two layers. The first layer is for AE signal modeling in normal conditions using the proposed hybrid Gaussian support vector autoregressive Laguerre technique, while the subsequent layer is used for AE signal estimation using the proposed hybrid fuzzy strict-feedback backstepping observer. The proposed hybrid strict-feedback backstepping digital twin is formed to overcome the nonstationary and nonlinear behavior of the AE bearing signals. Moreover, a residual signal generator is used to obtain the difference between the estimated and original AE signals. A machine learning crack size/type diagnosis that includes resampling and RMS feature extracting is used in combination with a support vector machine for the diagnosis of different bearing defects with various levels of severities. A bearing dataset containing normal conditions and seven fault severities was used to validate the proposed algorithm. It was observed that the proposed hybrid strict-feedback backstepping digital twin was able to estimate the normal and abnormal signals, which resulted in a superior diagnostic performance of the proposed model for both fault pattern and crack size identification. Moreover, the proposed hybrid strict-feedback backstepping digital twin was compared to two fault diagnosis algorithms (i.e., *ALSGL*-*SBI* and *ALSGL*-*SB*). The results demonstrate that the proposed algorithm was more effective than the other methods regardless of the nonlinearity contained in the AE signals due to multiple fault severities. However, in the case of the identification of inner–ball faults, the performance of the proposed method slightly depreciated, which underscores the need for more complex classification algorithms in future work which could eventually result in superior diagnostic performance. In future work, combining deep-learning and observation techniques will be studied to improve the reliability and robustness of digital twins.

## Figures and Tables

**Table 1:** AE UIAI-Lab bearing signal information.

Classes	Motor Speed (RPM)	Crack Sizes (mm)
HC	300, 400, 450 and 500	-
OC	300, 400, 450 and 500	3 and 6
IC	300, 400, 450 and 500	3 and 6
BC	300, 400, 450 and 500	3 and 6
IOC	300, 400, 450 and 500	3 and 6
IBC	300, 400, 450 and 500	3 and 6
OBC	300, 400, 450 and 500	3 and 6
IOBC	300, 400, 450 and 500	3 and 6

**Table 2:** Data acquisition system information [36].

AE Sensor (PAC WSα) [41]	PCI Board with 2-Channel AE Sensor [42]
Peak sensitivity (V/μbar): −62 dB	18-bit 40 MHz A/D conversion
Operating frequency range: 100–900 kHz	AE input: 2 channels (a 10 M samples/s rate one
Directionality: ±1.5 dB	and a 5 M samples/s one, as two channels
Resonant frequency: 650 kHz	were simultaneously used)

**Table 3:** Training and testing samples for RMS residual signals for signal classification using the SVM.

States	Training Samples (Numbers)	Testing Samples (Numbers)
Crack Type Diagnosis		
HC	300	100
OC	600	200
IC	600	200
BC	600	200
IOC	600	200
IBC	600	200
OBC	600	200
IOBC	600	200
**Crack Size Diagnosis (OC, IC, BC, IOC, IBC, OBC and IOBC)**		
3 mm	300	100
6 mm	300	100

**Table 4:** Average accuracies of crack type diagnosis using the combined *ALSG*L-*SB* and SVM, *ALSGL*-*SBI* and SVM, and PDT and SVM approaches.

Conditions	*ALSGL*-*SB* and SVM (%)	*ALSGL*-*SBI* and SVM (%)	PDT and SVM (%)
HC	100	100	100
BC	88	92	98
IC	89	90	96
OC	83	91	96
IBC	85	89	94
IOC	89	89	97
OBC	84	90	98
IOBC	80	92	98
Average	87.25	91.63	97.13

**Table 5:** Average accuracies for crack size diagnosis using the combined *ALSGL*-*SB* and SVM, *ALSGL*-*SBI* and SVM, and PDT and SVM approaches.

Fault Type	Crack Sizes (mm)	*ALSGL*-*SB* and SVM (%)	*ALSGL*-*SBI* and SVM (%)	PDT and SVM (%)
IC	3	80	87	96
	6	84	91	98
OC	3	86	89	97
	6	90	92	98
BC	3	82	90	96
	6	85	89	96
IBC	3	83	88	98
	6	84	91	98
IOC	3	81	90	96
	6	80	90	94
OBC	3	80	89	95
	6	81	89	98
IOBC	3	82	88	98
	6	85	92	98
Average		83.1	89.7	96.9

## Data Availability

The data presented in this study are available on request from the corresponding author.
